# Bismuth nitrate pentahydrate-induced novel nitration of eugenol

**DOI:** 10.1186/2191-2858-1-9

**Published:** 2011-09-20

**Authors:** Luis Canales, Debasish Bandyopadhyay, Bimal K Banik

**Affiliations:** 1 Department of Chemistry, The University of Texas-Pan American, 1201, West University Drive, Edinburg, TX 78539, USA

## Abstract

**Background:**

Eugenol, the main constituent of clove oil possesses a number of medicinal activities. To enhance the medicinal property, structural modification is required. On the other hand, bismuth nitrate pentahydrate has been established as an excellent eco-friendly nitrating agent for several classes of organic compounds.

**Results:**

Bismuth nitrate pentahydrate-induced nitration of eugenol has been investigated very thoroughly. Twenty five different conditions have been studied. The microwave-induced solvent-free reaction has been identified as the best condition.

**Conclusions:**

Spectral analyses confirm that 5-nitroeugenol is the sole product in all the cases. No oxidized or isomerized product could be detected.

## Background

*Syzygium aromaticum *L., popularly known as clove, belongs to the plant family Myrtaceae, and has been used in folk medicine and dental treatment. Eugenol (4-allyl-2-methoxyphenol), the main component of clove oil, is an allyl chain-substituted guaiacol in the biosynthesized phenylpropanoid compound class derived from *Syzygium aromaticum *L. and widely used in medicine[1]. It is widely cultivated in India, Indonesia, Sri Lanka, Madagascar, and Brazil. In addition, it is commonly used in root canal and temporary fillings; it shows antibacterial activity, and helps in dental caries treatment and periodontal disease[2,3]. Clove oil has been successfully used for some breath problems[3]. It is slightly soluble in water and soluble in organic solvents. A recent report[4] reveals the insecticidal effect of eugenol. Anti-inflammatory and antinociceptive activities of eugenol have also been reported[5]. Moreover, eugenol is reported to possess antioxidant and anticancer properties[6].

In order to study the biological activities of eugenol derivatives, nitration by conventional nitric acid-sulfuric acid or a nitronium tetrafluoborate method were performed. These reactions require a mixture of concentrated or fuming nitric acid with sulfuric acid leading to excessive use of hazardous chemicals[7]. Nitration of eugenol and its derivatives was reported using HNO_3_/H_2_SO_4_[8] or by HNO_3_/Et_2_O[9]. In addition, difficult work-up procedure and low yield were also observed as a result of some other side reactions.

On the other hand, the usefulness of clay-mediated organic synthesis has been documented in a large number of publications which includes Michael addition [10], regioselective synthesis of carbazoles [11], selective hydrolysis of nucleosides [12], and Knoevenagel/hetero Diels-Alder reaction [13]. We have demonstrated the use of trivalent bismuth nitrate pentahydrate in organic synthesis. These experiments have resulted in various methods that include protection of carbonyl compounds [14], Michael reaction [15], nitration of aromatic systems [16], deprotection of oximes and hydrazones [17], and Paal-Knorr synthesis of pyrroles [18]. Our success in the bismuth nitrate-induced reaction has revealed [19] that this reagent acts as a Lewis acid.

We have been studying metal/metal salts-mediated reactions with the aim of developing several biologically active compounds; including anticancer polyaromatic compounds [20] and anticancer β-lactams [21]. Toward this goal, we also demonstrated that an effective bismuth nitrate-mediated nitration of polycyclic aromatic hydrocarbons. We reported the nitration of estrone with metal salts which exclusively depends on the nature of the solid surfaces [22]. Herein we report the direct nitration of eugenol using bismuth nitrate pentahydrate, an economical, easily available and eco-friendly salt. A comparison with various solvents, solid supports along with solvent-free condition has been carried out.

## Results

**Figure 1:**
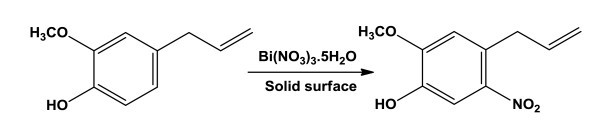
**Nitration of eugenol with bismuth nitrate on solid surface**.

In previous work we reported the nitration of estrone with different types of metal salts in the presence of solid surfaces under various conditions[22]. It has been clearly established that the nitration reaction induced by metal salts depend on the nature of the solid surface, nitrating agents, and reaction conditions. We have extensively studied the nitration of eugenol using various methods and solid surfaces (Figure 1). The results are summarized in Table 1.

**Table 1:** Bismuth nitrate-induced nitration of eugenol under different conditions

Entry	Solid surface	Method/Solvent	Yield (%)
1	Florisil	Dean-Stark/Benzene	75

2	Silica gel	Dean-Stark/Benzene	100

3	Molecular sieves	Dean-Stark/Benzene	80

4	KSF clay	Dean-Stark/Benzene	95

5	Neutral alumina	Dean-Stark/Benzene	90

6	Florisil	Reflux/DCM	80

7	Silica gel	Reflux/DCM	90

8	Molecular sieves	Reflux/DCM	80

9	KSF clay	Reflux/DCM	92

10	Neutral alumina	Reflux/DCM	84

11	Florisil	Dry	NR

12	Silica gel	Dry	NR*

13	Molecular sieves	Dry	NR

14	KSF clay	Dry	NR

15	Neutral alumina	Dry	NR

16	Florisil	Microwave/Solvent Free	90

17	Silica gel	Microwave/Solvent Free	100

18	Molecular sieves	Microwave/Solvent Free	95

19	KSF clay	Microwave/Solvent Free	100

20	Neutral alumina	Microwave/Solvent Free	92

21	Florisil	Reflux/Benzene	83

22	Silica gel	Reflux/Benzene	100

23	Molecular sieves	Reflux/Benzene	85

24	KSF clay	Reflux/Benzene	95

25	Neutral alumina	Reflux/Benzene	92

*No reaction

## Discussion

Bismuth nitrate pentahydrate is the metal nitrate used in this experimentation process, although the effect of many others such as CAN, Zn(NO_3_)_2_, Ca(NO_3_)_2_, LaNO_3_, NaNO_3_, and Cu(NO_3_)_2 _were also studied elsewhere. Bismuth nitrate pentahydrate was confirmed as the best nitrating agent among all others. Dry conditions and solvent-free methods along with commercial solvents without any purification were investigated in order to identify the best conditions for this reaction. Reactions were performed at high temperature using Dean-Stark water separator, traditional reflux, and conventional kitchen microwave-induced methods. Solid surfaces such as florisil, silica gel, molecular sieves, KSF clay, and neutral alumina were used as solid support in the reaction. It was discovered that silica gel is the best solid surface. In some cases (entries 2, 17 and 22), the reaction gave 100% yield of the product (4-allyl-2-methoxy-5-nitrophenol). Eugenol and bismuth nitrate along with KSF clay as solid support, under the conventional microwave and solvent-free condition produced 100% yield (entry 19). Quantitative yield was also observed under reflux in benzene with bismuth nitrate in presence of silica gel (entry 22). No reaction was observed when Bi(NO_3_)_3 _was used at room temperature even after 24 h (entries 11-15).

## Conclusions

In conclusion, metal nitrate-induced nitration of eugenol has been successfully carried out under various conditions and the formation of a single product (4-allyl-2-methoxy-5-nitrophenol) has been observed in variable yields. The exploratory results described herein confirm that bismuth nitrate pentahydrate is the reagent of choice in the absence of any solvent under microwave-irradiation condition (entry 19). Importantly, in contrast with nitric acid-mediated method, these reactions mediated by bismuth nitrate have several important characteristics. For example, no isomerization of the alkene moiety has been observed, regioselectivity remains identical irrespective of the solid supports and conditions, no oxidation of the alkene/aromatic systems has been observed, and phenolic hydroxyl group has no influence on the regioselectivity of the reactions. On the basis of these important and selective observations, this method will find very useful applications in synthetic chemistry of electrophilic aromatic nitration reaction.

## Methods

### General

FT-IR spectra were registered on a Bruker IFS 55 Equinox FTIR spectrophotometer as KBr discs. ^1^H-NMR (600 MHz) and 13C-NMR (125 MHz) spectra were obtained at room temperature with Bruker-600 equipment using TMS as internal standard and CDCl_3 _as solvent. Analytical grade chemicals (Sigma-Aldrich Corporation) were used throughout the project. Deionized water was used for the preparation of all aqueous solutions.

### General procedure for the nitration of Eugenol

In general, eugenol (1 mmol) and bismuth nitrate pentahydrate (1 eqv.) were mixed and the mixture was studied under different conditions varying the method, solid support and/or solvent as mentioned in Table 1. A representative experimental procedure (entry 2) is as follows: Eugenol (1 mmol) and silica gel (500 mg) was added to a suspension of bismuth nitrate pentahydrate (1 eqv.) in dry benzene (20 mL). The mixture was refluxed using Dean-Stark water separator for 2 h. The progress of the reaction was monitored by TLC. The reaction mixture was then repeatedly extracted (3 × 10 mL) with dichloromethane, washed with saturated solution of sodium bicarbonate, brine and water successively. The organic layer was dried over anhydrous sodium sulfate and concentrated to afford the crude product which was purified by column chromatography (silica gel, hexane/ethyl acetate).

#### 4-Allyl-2-methoxy-5-nitrophenol

sticky mass; IR (KBr disc, cm^-1^): 2369, 1522, 1457, 1243, 1136, 1061, 941, 810 and 712; ^1^H NMR (CDCl_3_, 600 MHz) δ: 10.67 (s, 1 H), 7.45 (s, 1 H), 6.84 (s, 1 H), 5.89 (m, 1 H), 5.05 (m, 2 H), 3.83 (s, 3 H), 3.27 (d, 2 H, *J *= 1.1 Hz). ^13^C NMR (CDCl_3_, 125 MHz) δ: 149.86, 144.88, 135.94, 133.64, 132.46, 128.63, 127.43, 125.07, 56.70, 36.74.

## Competing interests

The authors declare that they have no competing interests.
